# Defining research priorities and needs in cancer symptoms for adults diagnosed with cancer: an Australian/New Zealand modified Delphi study

**DOI:** 10.1007/s00520-023-07889-y

**Published:** 2023-07-03

**Authors:** Vanessa M. Yenson, Ingrid Amgarth-Duff, Linda Brown, Cristina M. Caperchione, Katherine Clark, Andrea Cross, Phillip Good, Amanda Landers, Tim Luckett, Jennifer Philip, Christopher Steer, Janette L. Vardy, Aaron K. Wong, Meera R. Agar

**Affiliations:** 1 University of Technology Sydney, Sydney, NSW Australia; 2 IMPACCT (Improving Palliative, Aged and Chronic Care Through Clinical Research and Translation), University of Technology Sydney, Sydney, NSW Australia; 3 Cancer Symptom Trials (CST), IMPACCT, University of Technology Sydney, Sydney, NSW Australia; 4 Telethon Kids Institute, Perth, WA Australia; 5 Palliative Care Clinical Studies Collaborative (PaCCSC), IMPACCT, University of Technology Sydney, Sydney, NSW Australia; 6 School of Sport, Exercise and Rehabilitation, University of Technology Sydney, Sydney, NSW Australia; 7 CST Management Advisory Committee, IMPACCT, University of Technology Sydney, Sydney, NSW Australia; 8 Northern Sydney Local Health District Supportive and Palliative Care Network, St Leonards, Sydney, NSW Australia; 9 Northern Clinical School, The University of Sydney, St Leonards, Sydney, NSW Australia; 10 Northern Sydney Cancer Centre, Royal North Shore Hospital, St Leonards, Sydney, NSW Australia; 11 Consumer Advocate, Cancer Symptom Trials, IMPACCT, University of Technology Sydney, Sydney, NSW Australia; 12 CST Scientific Advisory Committee, Cancer Symptoms Trials, IMPACCT, University of Technology Sydney, Sydney, NSW Australia; 13 Palliative and Supportive Care, Mater Misericordiae, South Brisbane, QLD Australia; 14 Department of Palliative Care, St Vincent’s Private Hospital, Brisbane, QLD Australia; 15 Mater Research – University of Queensland, South Brisbane, QLD Australia; 16 Palliative Care, Department of Medicine, University of Otago, Christchurch, New Zealand; 17 University of Melbourne, Palliative Medicine, Melbourne, VIC Australia; 18 Peter MacCallum Cancer Centre, Palliative Care, Melbourne, VIC Australia; 19 Royal Melbourne Hospital, Melbourne, VIC Australia; 20 University of New South Wales Rural Clinical Campus, Albury-Wodonga, NSW Australia; 21 Border Medical Oncology, Albury-Wodonga Regional Cancer Centre, Albury-Wodonga, NSW Australia; 22 Centre for Medical Psychology and Evidence-Based Decision-Making, University of Sydney, Sydney, NSW Australia; 23 Concord Cancer Centre, Concord Repatriation General Hospital, Sydney, NSW Australia; 24 Faculty of Medicine and Health, University of Sydney, Sydney, NSW Australia

**Keywords:** Neoplasms, Cancer symptoms, Delphi, Consumers, Healthcare professionals

## Abstract

**Purpose:**

This study asked consumers (patients, carers) and healthcare professionals (HCPs) to identify the most important symptoms for adults with cancer and potential treatment interventions.

**Methods:**

A modified Delphi study was conducted involving two rounds of electronic surveys based on prevalent cancer symptoms identified from the literature. Round 1 gathered information on participant demographics, opinions and/or experience on cancer symptom frequency and impact, and suggestions for interventions and/or service delivery models for further research to improve management of cancer symptoms. In Round 2, respondents ranked the importance of the top ten interventions identified in Round 1. In Round 3, separate expert panels of consumers and healthcare professionals (HCPs) attempted to reach consensus on the symptoms and interventions previously identified.

**Results:**

Consensus was reached for six symptoms across both groups: fatigue, constipation, diarrhoea, incontinence, and difficulty with urination. Notably, fatigue was the only symptom to reach consensus across both groups in Round 1.

Similarly, consensus was reached for six interventions across both groups. These were the following: medicinal cannabis, physical activity, psychological therapies, non-opioid interventions for pain, opioids for breathlessness and cough, and other pharmacological interventions.

**Conclusions:**

Consumers and HCPs prioritise differently; however, the symptoms and interventions that reached consensus provide a basis for future research. Fatigue should be considered a high priority given its prevalence and its influence on other symptoms. The lack of consumer consensus indicates the uniqueness of their experience and the need for a patient-centred approach. Understanding individual consumer experience is important when planning research into better symptom management.

**Supplementary Information:**

The online version contains supplementary material available at 10.1007/s00520-023-07889-y.

## Introduction

In 2022, approximately 162,000 Australians were diagnosed with cancer [1]. While the immediate priority for many patients is cure, in those where this is not possible, the aim is to achieve remission, prolong survival, and/or improve quality of life. However, many patients experience symptoms related to their disease and/or its treatment (collectively referred to as ‘cancer symptoms’), with common problems including psychosocial concerns, fatigue, sleep disturbance, weakness, pain, weight changes, sexual dysfunction, and cognitive impairment [2–9].

Over time, cancer symptoms can change as patients continue on their journey. Toxicities from cancer treatments range from acute and severe [10–12] to chronic [13–15]. Cancer symptoms can persist over acute, survivorship and palliative stages, exacerbating fatigue, weakness, gastrointestinal, cognitive, and sexual functioning [2–5, 7, 16]. Clinicians have a responsibility to prevent, detect, and manage ongoing or new cancer symptoms, across the survivorship continuum [16–20].

Consumers (defined as patients and carers), clinicians, and researchers have identified the need for consolidated efforts to improve the evidence base underpinning cancer symptom management and to implement models of symptomatic and supportive care into practice. To achieve this goal, a coherent and focused effort needs to be applied to identify research priorities and, in turn, to improve outcomes for people affected by cancer. Those symptoms that persist, or even worsen over time must be identified and categorised—and research, resources, and quality randomised clinical trials should be directed to those undermanaged symptoms to provide evidence for future clinical improvement. Lastly, identification of new symptoms—expected or unexpected—will facilitate future surveillance, prevention, detection, and management to improve every cancer survivor’s quality of life.

Previous Australian studies have focused on determining research priorities in psycho-oncology [21, 22], or adult palliative care [23], but there remains a lack of clear guidance in Australia and New Zealand as to where resources might be best invested for cancer symptom prevention, detection, and treatment. Prior studies to set priorities for cancer research have used a Delphi-based approach to answer questions where evidence is sometimes lacking [24].

Given the current lack of consensus, the purpose of this modified Delphi study was to identify the most important symptoms for people diagnosed with cancer, to establish those symptoms that require further support and research, and to identify potential interventions to manage these.

## Methods

### Study design

The Delphi technique is a reliable, iterative method, used since the 1950s [25], which aims to achieve consensus among a group of experts on a defined clinical problem, using open and closed-ended questions [26, 27]. Each sequential Delphi round is refined based on feedback received in the previous round. In this study, a modified Delphi process was followed to provide a focus for directing further resources, research, and clinical trials to support people diagnosed with cancer. The Delphi was modified by administering the first two rounds as online surveys (‘e-Delphis’) [25], which ensured anonymity and reduced the potential for group-think (influence by group opinions) [28]. Participants in Round 1 were given the option to provide their email address to take part in subsequent rounds. The third round was conducted as virtual consensus meetings. Although participants were likely to have completed all three rounds, new participants could join Round 2 as this was available through the CST Projects Website. This study is reported in accordance with the Guidance on Conducting and Reporting Delphi Studies (CREDES) guidelines [24].

### Expert panels

The consumer panel included people diagnosed with cancer and their support people (collectively referred to as ‘consumers’). The healthcare professional (HCPs) panel included palliative care medical specialists (oncologists, palliative care specialists, haematologists), nurses, and allied health professionals such as physiotherapists and social workers.

Purposive [29] and snowballing [30] techniques were used to sample for both groups of participants including email invitations circulated via professional organisation mailing lists, postings on cancer patient support websites, mailing lists, and social media pages including Twitter (see the ‘Acknowledgements’ section for a full list of organisations).

The study was approved by the University of Technology Sydney’s (UTS) Human Research Ethics Committee (ETH19-4484, ETH21-6079, and ETH21-6558). All respondents provided electronic informed consent and did not receive compensation for their participation.

### Delphi process

**Fig. 1:**
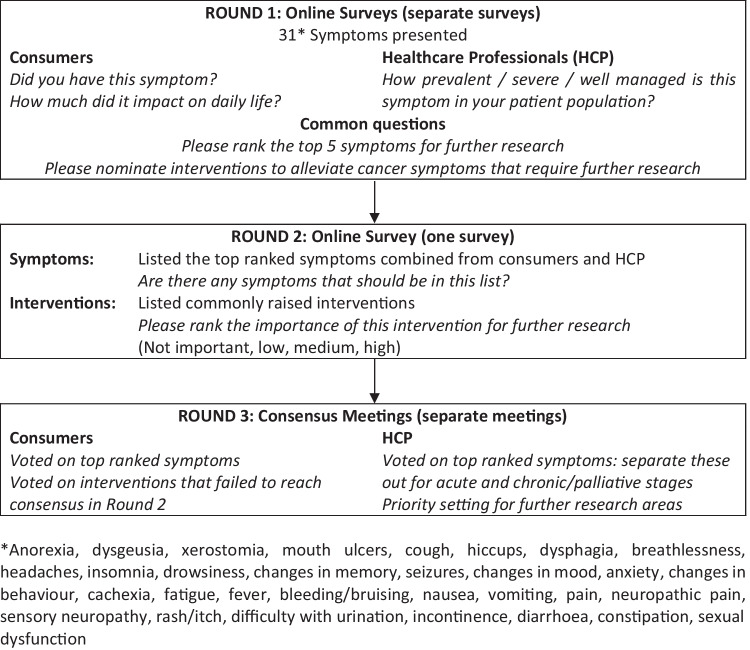
Delphi process

The process used in this modified Delphi study is shown in Fig. 1. The first two rounds of the Delphi study were conducted as online surveys with HCPs and consumers (Fig. 1).

#### Survey Round 1

This comprised three main Delphi elements [31]: (1) participant demographics, (2) opinion and/or experience with cancer symptom frequency and impact on daily life during and after cancer treatment, and (3) suggestions for pharmacological/non-pharmacological interventions and/or service delivery models requiring further research to improve cancer symptom management.

Thirty-one symptoms (Table 3), chosen from previous literature, expert clinical advice, and clinical and consumer experience, were presented [4–7, 32]. Consumers were asked to rate their experience of these symptoms, while HCPs were asked to consider their clinical experience and rate the prevalence, severity, and success at managing patient symptoms on a 4-point Likert scale. Participants had the opportunity to nominate cancer symptoms that were not already listed, and to rank the symptoms according to research priority.

Participants were asked to suggest interventions by free text responses, with guidance given to ‘list medications (for example a specific drug to manage a specific symptom), medical devices (for example a device which improved muscle strength), or other non-drug treatments (e.g. physical activity to reduce pain due to nerve damage)’.

Results from both Rounds 1 and 2 were disseminated to the Cancer Symptom Trials (CST) Management Advisory Committee for review and comment prior to the next round.

#### Survey Round 2

Round 2 comprised of three sections: (1) demographic information, (2) top ten cancer symptoms ranked from Round 1, and (3) rankings on the importance of each symptom intervention for further research on a 4-point Likert scale (not important, low, medium, high). Free text fields allowed participants to suggest other interventions and provide suggestions for discussion at follow-up consensus meetings.

#### Consensus meetings

HCPs and consumers were invited to attend separate 1-hour consensus meetings via video conference. This was to ensure that the consumers were not intimidated by the presence of HCPs and could freely express themselves.

Participants were presented with the responses to Rounds 1 and 2, including (1) proposed priority areas for inclusion, (2) a summary of symptoms and interventions that did not reach a consensus, and (3) newly suggested symptoms and interventions.

In the consensus meeting, HCPs requested separate survey to differentiate important symptoms between the acute (active treatment) and chronic (survivorship or palliative) stages, which was completed as online surveys.

Participants discussed the symptoms and interventions that required refinement and decided whether further symptoms needed to be included. This informed the final consensus list of research priorities and questions to directly tackle inadequately managed cancer symptoms in adults diagnosed with cancer.

#### Interpretation and data analysis

All data were de-identified prior to analysis. Responses to the *Round 1* survey were analysed descriptively using frequencies and percentages. For consumers, consensus was reached if ≥ 70% experienced the symptom (‘prevalence’) and ≥ 70% classified these as having moderate or major impact (‘impact’). For HCP, consensus was reached if ≥ 70% rated the symptom as prevalent, severe, and undermanaged in their patient population.

In *Round 2*, descriptive statistics were computed, and the percentages of agreement of the responses were generated. Given that a Delphi consensus parameter can vary anywhere from 51 to 100%, we considered consensus to be reached when ≥ 70% of participants indicated a response in the higher priority categories (‘medium’ or ‘high’ on a 4-point scale) [25, 33]. Mean rankings were given to each research topic, calculated from weighted scores (so that a first ranking was given a weighted score of four, a second ranking a weighted score of three, etc.). An analysis of variance examined differences in weighted ranking scores by participant group (consumer, HCP).

Data from the Consensus meetings were summarised in detailed minutes, with consensus on key cancer symptoms agreed upon at the conclusion of the meeting. To ensure external validity, the final draft of the resulting guidance was reviewed and approved by the UTS CST Management Advisory Committee prior to publication and dissemination.

## Results

### Round 1

A Delphi was conducted between 01 June 2020 and 31 August 2020. It was completed by 332 consumers (Table 1) and 51 HCPs (Table 2). Round 1 surveys took approximately 30 min to complete.

**Table 1:** Consumer demographics (all rounds)

	Round 1 (*n* = 615)	Round 2 (*n* = 63)	Consensus building (*n* = 9)
Country			
Australia	506 (82.3%)	61 (96.8%)	8 (88.9%)
New Zealand	42 (6.8%)	2 (3.2%)	1 (11.1%)
*Missing*	67 (10.9%)	-	
Australian state/territory			
NSW	189 (30.7%)	22 (34.9%)	2 (22.2%)
VIC	117 (19.0%)	16 (25.4%)	2 (22.2%)
QLD	86 (14.0%)	10 (15.9%)	2 (22.2%)
SA	33 (5.4%)	5 (7.9%)	2 (22.2%)
WA	34 (5.5%)	1 (1.6%)	-
TAS	19 (3.1%)	2 (3.2%)	-
ACT	27 (4.4%)	4 (6.3%)	-
NT	1 (0.2%)	1 (1.6%)	-
*Missing*	109 (17.7%)	-	-
Cancer type*			
Bone	13 (2.1%)	-	-
Brain/central nervous system	78 (12.7%)	5 (7.9%)	-
Breast	192 (31.2%)	24 (38.1%)	1 (11.1%)
Genitourinary	1 (0.2%)	-	-
Gynaecological	23 (3.7%)	3 (4.8%)	1 (11.1%)
Haematological	34 (5.5%)	8 (12.7%)	1 (11.1%)
Head and neck	21 (3.4%)	4 (6.3%)	1 (11.1%)
Hepatobiliary	5 (0.8%)	1 (1.6%)	-
Kidney	5 (0.8%)	-	-
Lower GI	22 (3.6%)	3 (4.8%)	1 (11.1%)
Lung	41 (6.7%)	2 (3.2%)	-
Skin	68 (11.1%)	8 (12.7%)	1 (11.1%)
Thyroid	1 (0.2%)	1 (1.6%)	-
Upper GI	-	1 (1.6%)	-
Pancreatic	20 (3.3%)	1 (1.6%)	1 (11.1%)
Prostate	75 (12.2%)	8 (12.7%)	2 (22.2%)
Consumer background			
Person living with cancer	440 (71.5%)	59 (93.7%)	7 (77.8%)
Spouse of person living with cancer	49 (8.0%)	3 (4.8%)	2 (22.2%)
Child of person living with cancer	10 (1.6%)	1 (1.6%)	-
Caregiver	28 (4.6%)	-	-
Other	22 (3.6%)	-	-
*Missing*	66 (10.7%)	-	-
Age (years), mean (SD)	54.5 (12.5) *n* = 542		-
Treatments received			
Surgery	396 (64.4%)	48 (76.2%)	8 (88.9%)
Chemotherapy	313 (50.9%)	42 (66.7%)	8 (88.9%)
Radiation	289 (47.0%)	36 (57.1%)	5 (55.6%)
Hormone therapy	163 (26.5%)	10 (15.9%)	-
Immunotherapy	91 (14.8%)	11 (17.5%)	2 (22.2%)
Other	67 (10.9%)		2 (22.2%)

^*^May not sum to 100% as patients may have had more than one diagnosis

**Table 2:** Health care professional demographics (all rounds)

	Round 1 (*n* = 133)	Round 2 (*n* = 13)	Consensus building (*n* = 11)
Country			
Australia	89 (66.9%)	10 (77%)	10 (90.0%)
New Zealand	30 (22.6%)	3 (23%)	1 (9.1%)
*Missing*	14 (10.5%)		-
Australian state/territory			
NSW	33 (24.8%)	3 (23.1%)	5 (45.5%)
VIC	26 (19.5%)	-	4 (36.4%)
QLD	12 (9.0%)	3 (23.1%)	1 (9.1%)
SA	3 (2.3%)	-	-
WA	8 (6.0%)	1 (7.7%)	-
TAS	6 (4.5%)	1 (7.7%)	-
ACT	1 (0.8%)	-	-
NT	-	-	-
*Missing*	44 (33.1%)	2 (15.4%)	-
Cancer type*			
Bone	55 (41.4%)	8 (61.5%)	4 (36.4%)
Brain/central nervous system	68 (51.1%)	8 (61.5%)	9 (81.8%)9
Breast	76 (57.1%)	10 (76.9%)	6 (54.5%)
Genitourinary	71 (53.4%)	10 (76.9%)	7 (63.6%)
Gynaecological	65 (48.9%)	9 (69.2%)	7 (63.6%)
Haematological	60 (45.1%)	11 (84.6%)	9 (81.8%)
Head and neck	63 (47.4%)	10 (76.9%)	6 (54.5%)
Hepatobiliary	68 (51.1%)	10 (76.9%)	9 (81.8%)
Kidney	61 (45.9%)	10 (76.9%)	6 (54.5%)
Lower GI	78 (58.6%)	12 (92.3%)	8 (72.7%)
Lung	75 (56.4%)	12 (92.3%)	10 (90.9%)
Skin	56 (42.1%)	11 (84.6%)	6 (54.5%)
Thyroid	45 (33.8%)	6 (46.2%)	3 (27.3%)
Upper GI	78 (58.6%)	11 (84.6%)	10 (90.9%)
Area of expertise			
Medical	51 (38.3%)	9 (69.2%)	10 (90.0%)
Research	6 (4.5%)	3 (23.1%)	1 (9.1%)
Nursing	53 (39.8%)	1 (7.7%)	-
Allied health	8 (6.0%)	-	-
Care coordinator	1 (0.8%)	-	-
*Missing*	14 (10.5%)		-
Medical discipline*			
Palliative medicine	45 (33.8%)	6 (46.2%)	6 (54.5%)
Medical oncology	5 (3.8%)	2 (15.4%)	5 (45.5%)
Radiation oncology	1 (0.8%)	1 (7.7%)	-
*Missing*	82 (61.7%)	-	
Institution level**			
University	11 (8.3%)	4 (30.7%)	8 (72.7%)
Public hospital/healthcare entity	95 (71.4%)*	8 (61.5%)	9 (81.8%)
Private hospital/healthcare entity	17 (12.8%)*	6 (46.2%)	5 (45.5%)
Community or primary care	17 (12.8%)	2 (15.4%)	-
Other	5 (3.8%)		
Highest qualification			
Certificate	3 (2.3%)	1 (7.7%)	-
Diploma	12 (9.0%)	1 (7.7%)	-
Bachelor	43 (32.3%)	4 (30.7%)	5 (45.5%)
Masters	42 (31.6%)	2 (15.4%)	-
PhD	3 (2.3%)	5 (38.5%)	6 (54.5%)
Other	4 (3.0%)		-
*Missing*	14 (10.5%)		-
Length of time working with cancer patients	2.5 (0.7) years		
0–5 years	16 (12.0%)	-	-
5–10 years	22 (16.5%)	3 (23.1%)	2 (18.2%)
10 + years	81 (60.9%)	10 (77%)	9 (81.8%)
Cancer treatments offered at facility			
Cancer surgery	81 (60.9%)	11 (84.6%)	8 (72.7%)
Chemotherapy	93 (69.9%)	11 (84.6%)	9 (81.8%)
Radiotherapy	79 (59.4%)	11 (84.6%)	9 (81.8%)
Immunotherapy	85 (63.9%)	11 (84.6%)	9 (81.8%)
Targeted therapy	81 (60.9%)	11 (84.6%)	9 (81.8%)
Bone marrow transplant	-	6 (46.2%)	4 (36.4%)
Other	32 (24.1%)	-	3 (27.3%)

^*^May sum to more than 100% as clinicians may work with more than one type of cancer, or at more than one type of institution. **102 (76.7%) HCPs worked across both public and private hospitals

#### Consumers

**Fig. 2:**
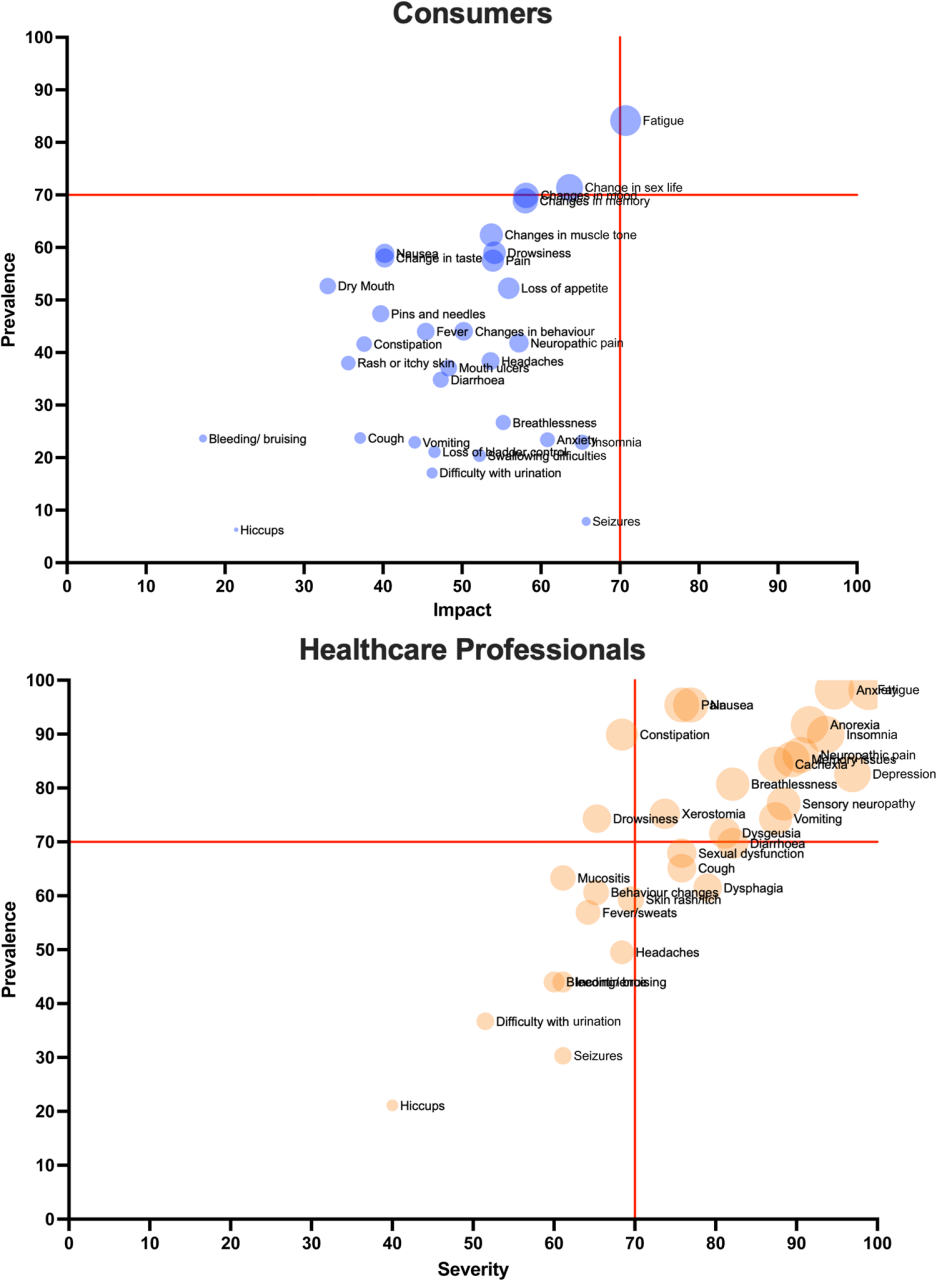
Prevalence and impact of cancer symptoms rated by consumers (blue) and health care professionals (orange)

Most consumers were patients rather than support people and had received a cancer diagnosis at some point in time (71.5%; Table 1). The prevalence and impact of symptoms reported by consumers (Fig. 2 and Supplemental Table 1) found fatigue to be the only symptom to reach consensus for further research in Round 1 (Table 3, Supplemental Table 2). A range of other physical, gastrointestinal, and mental health symptoms was identified via free text responses.

**Table 3:**
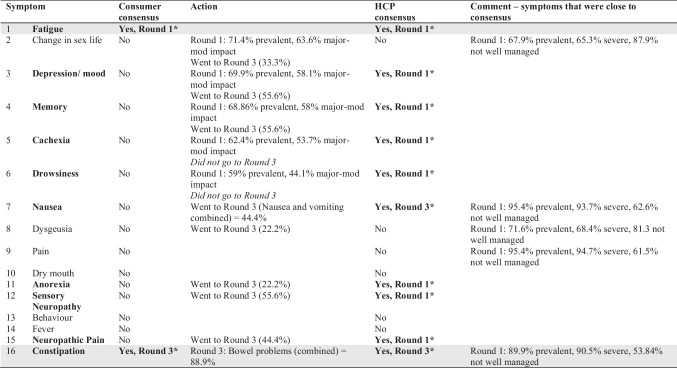

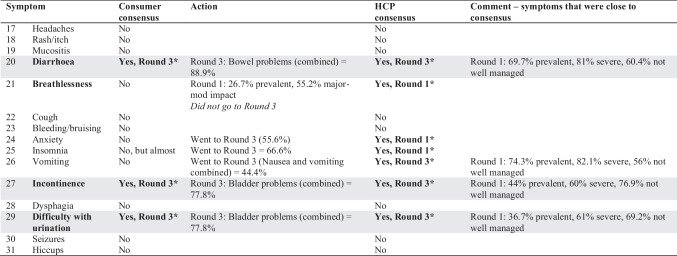
Symptom consensus across all rounds (consumer and HCP). Symptoms which reached consensus are marked in bold and with an asterisk. Shading shows consensus reached across both consumer and HCP groups

Certain pharmacological (e.g. medicinal cannabis) and non-pharmacological priorities (e.g. physical exercise) were raised by consumers as research intervention priorities (Table 3). Other aspects of care were also raised, such as communication with clinicians.

#### Healthcare professionals

HCP (*n* = 133) were palliative medicine specialists (38%) or oncology nurses (40%) working in the hospital setting (77%), with over 10-year experience (61%; Table 1). The prevalence, severity, and management of symptoms reported by HCPs (Fig. 2 and Supplemental Table 2) that reached consensus were as follows: fatigue, anxiety, anorexia, insomnia, neuropathic pain, memory, cachexia, depression/mood, breathlessness, sensory neuropathy, and drowsiness (Table 3).

HCPs named specific pharmacological agents (such as anamorelin for loss of appetite and mirtazapine for breathlessness) and non-pharmacological interventions (such as psychological therapies) that warrant further research as treatments for cancer symptoms (Table 3).

### Round 2

Free-text responses from Round 1 were analysed thematically by two researchers and included in the Round 2 survey. Researchers discussed any uncertainties about the codes until an agreement was met. These coded themes were then employed in the Round 2 survey. This second Delphi survey was conducted between 08 July 2021 and 30 September 2021. It was completed by 63 consumers and 13 HCPs. Participant demographics are reported in Tables 1 and 2.

#### Consumers

Consumers from Round 1 who were willing to participate in Round 2 were invited by email to participate in Round 2. Of the 63 consumers (Table 1), most had cancer (94%) and lived in Australia (97%). Like in Round 1, most had experienced breast cancer (38%), followed by blood, skin, and prostate cancers (all 12.7%). Additional symptoms identified during this round are listed in Table 3.

The consumers subsequently rated the interventions that reached consensus in Round 1. These were the following: future research into medicinal cannabis, physical activity, psychological therapies, vitamin D, non-opioid interventions for pain, opioids for breathlessness, and pharmacological interventions for depression and anxiety (Table 4). Most consumers felt they had insufficient knowledge about two complementary medicines (Murin Murin bush medicine (*Scaevola spinescens*) for nausea and fatigue, and *Boswellia serrata* for muscle atonia and brain swelling). As such, these complementary medicines were not re-presented in Round 3.

**Table 4:**
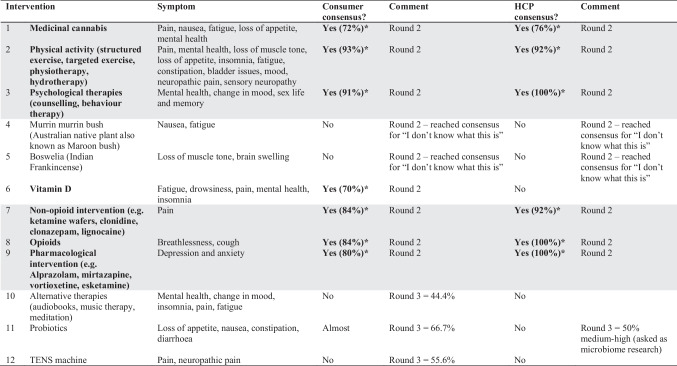
Intervention consensus across all rounds (consumer and HCP). Interventions reaching consensus are marked in bold and with an asterisk. Shading shows consensus reached across both consumer and HCP groups

#### Healthcare professionals

The majority of HCPs in Round 2 had previously completed Round 1 (10; 77%). Most were doctors (69%) and working in palliative medicine (46%). The majority were working in Australia (77%) in the hospital system (77%) and had more than 10-year experience (77%, Table 1).

HCPs raised nausea and vomiting (23%), constipation (8%), cachexia/weight loss (8%), dysphagia (8%), breathlessness (8%), itch (8%), hiccups (8%), and lymphoedema (8%) as additional noteworthy symptoms.

Of the interventions raised in Round 1, HCPs achieved consensus on suggesting further research into medicinal cannabis, physical activity, psychological therapies, non-opioid interventions for pain, opioid interventions for breathlessness, and pharmacological interventions for depression and anxiety (Table 4). Like the consumer group, HCPs indicated insufficient knowledge of the two complementary medicines, (*Scaevola spinescens* and *Boswellia serrata*) to recommend further research on these products. Other interventions proposed for further research included benzodiazepines for anxiety, breathlessness, or insomnia (*n* = 1), acupuncture as a general research area (*n* = 1), and rehabilitation for swallowing difficulties (*n* = 1).

### Consensus meeting

Consumer consensus meetings were held in October and November 2021 (*n* = 9) and included consumers who participated in Rounds 1 and 2. The HCP meeting was held in December 2021 (*n* = 10) and included participants from previous rounds and new participants from our networks. The participant demographics are reported in Table 1. For each meeting (consumer or healthcare professional), the results of the previous rounds were presented; however, the results of any prior consensus meetings were not discussed with the consumers or HCP.

#### Consumers

Consensus was reached on the importance of research into bowel symptoms (constipation and diarrhoea) and bladder symptoms (incontinence and difficulty with urination). Consensus was close but not reached for insomnia and probiotics both 67% (Table 3). Consensus was not reached on any of the interventions considered in Round 3 (Table 4).

#### Healthcare professionals

In Round 3, the following cancer symptoms reached consensus: nausea, constipation, diarrhoea, vomiting, incontinence, and difficulty with urination (Table 3). Again, consensus was not reached on any of the interventions considered in Round 3 (Table 4). During the consensus meeting, the HCPs noted the difficulty in teasing out the differences between acute and chronic cancer symptoms or between initial treatment and palliative needs. To better define the latter, an additional survey was sent out to ten HCPs to try and better delineate symptoms of importance in the initial treatment and palliative care phases. For cancers undergoing active treatment, the highest priorities were fatigue, anxiety, sensory neuropathy, and neuropathic pain. For advanced cancers in the chronic or palliative stages, the highest priorities were fatigue, neuropathic pain, depression, anxiety, and insomnia.

## Discussion

After three Delphi rounds, the consumers and HCPs reached consensus on the following cancer symptoms as foci for future research: fatigue, constipation, diarrhoea, incontinence, and difficulty with urination. However, it was notable that the only symptom that reached consensus in Round 1 was fatigue. This was independent of cancer type or treatment, strongly highlighting the importance of fatigue as the first candidate for future research.

This finding may be due to the relationships between fatigue and other cancer symptoms, such as sleep, appetite, mood, behaviour, sexual function, nausea, and pain perception, and this inter-relatedness may also have affected the consumer response [34, 35]. Unlike many other cancer symptoms, there are currently no pharmacological solutions with which to mitigate or alleviate this symptom [36]. It affects young and old, all sexes, and those in the acute as well as chronic and palliative stages of cancer, regardless of the cancer treatment received.

In Round 1, the only cancer symptom that the consumers reached consensus on was fatigue. One explanation is that although other symptoms are present and distressing (such as pain and anorexia), current symptom management adequately prevents and/or alleviates them (such as analgesia and dietary and exercise changes). An additional explanation is that people with cancer expect transient symptoms (such as nausea and bowel problems) and ‘put up with them’ because they are perceived as a sign that the treatment is working [37].

While different interventions for addressing cancer-related fatigue have their own sets of advantages and disadvantages, a recent meta-analysis of non-pharmacological interventions found that multimodal therapy, cognitive behaviour therapy, and qigong (an ancient Chinese exercise) were most effective [38]. This finding further supports the validity of our results, which showed that both consumers and HCPs are interested in further research into psychological therapies for cancer symptoms. There is already some evidence to show that psychological therapies can play a role in the management of fatigue, insomnia, fear of recurrence, altered cognition and concerns about disease impact on intimacy, sexual activity, employment, and finances [39].

The identification of bowel problems (constipation and diarrhoea) as cancer symptoms of interest was unsurprising with constipation frequently reported by patients [40]. While pharmacological interventions need to be optimised when treating constipation, there is currently no evidence for the effectiveness of non-pharmacological interventions such as diet [40] and exercise [41]. While most previous research has looked for associations between physical activity and cancer survivorship, there are several systematic reviews that have shown positive effects from physical activity on fatigue, depression, and quality of life [41, 42]. Physical activity can also have a role to play in the management of pain, insomnia, metabolic syndrome, cognitive impairment, and osteoporosis [39, 41].

In this study, there was considerable complexity in trying to combine the HCP and consumer data due to the differences in perceived needs. We also found that there were a diverse range of symptoms reported by the consumer population, reflecting the diversity of their needs in relation to symptom control. For example, one participant reported difficulties with sensory neuropathy in their feet which resulted in them being unable to go bushwalking, impacting their daily life. Thus, cancer symptom management needs to be personalised to the specific circumstances of the patient, their cancer, and its treatment [43]. This diversity increased further when we compared the prevalence and impact of cancer symptoms as identified by the consumers and HCPs (Fig. 2).

One of the strengths of our study was the involvement consumers. While this is not a new concept, being first suggested in the 1970s, it has become central to healthcare policy with the advent of ‘value based care’ [44]. The importance of capturing the consumer voice is evident in the disparity we found between the views of consumers and HCPs. However, there were also several limitations to our study: although the online nature of the survey may have limited participation from some sectors of the community (for example, culturally and linguistically diverse people, those with poorer literacy, and those without access to a computer or the internet), this approach did allow us to reach participants in states and regional areas that might have been excluded during the lockdown stages of the COVID-19 pandemic. Additionally, the fact that there were no individual reminders sent to complete the online surveys (due to their anonymous nature), the Round 1 survey took considerable time to complete. This time between surveys or meetings may have contributed to the attrition rate of participants between rounds. Although the intention of the Round 1 survey was to include bereaved carers, the wording of the questions was not interpreted in this light, which might have also contributed to the attrition rate. Finally, given that a Delphi consensus parameter can vary from 51 to 100% [45, 46], we pragmatically considered consensus as being 70%. It is not known whether changing this cut-off would alter the consumer interpretation of our study.

Our study shows that understanding the importance of various cancer symptoms for both consumers and HCPs is complex and nuanced. As noted earlier, our findings highlight the complexity in trying to combine consumer and HCP responses, when perceived needs are being considered against lived experience which varies with tumour type and illness stage. This is an important finding in itself—and one that needs to be further explored. Other studies have reported that the prevalence of symptoms in people with cancer is often underestimated by clinicians (47), and this may then be reflected in the priorities assigned to symptoms such as diagnosis and treatment. The question remains as to what emphasis should be placed on the respective choices of consumers and HCPs with respect to the most important symptoms.

## Conclusions

Fatigue should be considered a high priority for further research into cancer symptoms given its relationship to other symptoms and universal presence. While our findings show that consumers and HCPs prioritise different cancer symptoms, those symptoms and interventions that reached consensus provide a compelling launching point for future research to improve cancer symptom management. The lack of consumer consensus in identifying cancer symptoms indicates the importance of patient-centred care when managing cancer symptoms and the importance of patient voice when determining future research directions.

## Supplementary Information

Below is the link to the electronic supplementary material.

Supplementary file1 (DOCX 33 KB)

## Data Availability

Data from this study is available upon request to the authors, and following appropriate ethical approval.
