# Supplementary material for: Defining research priorities and needs in cancer symptoms for adults diagnosed with cancer: an Australian/New Zealand modified Delphi study
Source: Support Care Cancer. 2023 Jul 3;31(7):436. doi: 10.1007/s00520-023-07889-y (PMC10317881; doi:10.1007/s00520-023-07889-y)
Supplement: Supplementary file 1 — Supplementary file1 (DOCX 33 KB) [file 520_2023_7889_MOESM1_ESM.docx]

Supplemental Table 1: Prevalence and impact of symptoms reported by consumers in Round 1

| Symptom | N | Prevalence (%) | Major or Moderate impact (% of prevalence) | Mild impact (% of prevalence) | No impact (% of prevalence) |
| --- | --- | --- | --- | --- | --- |
| Fatigue | 517 | 84.14 | 70.7 | 22.6 | 6.8 |
| Change in sex life | 514 | 71.40 | 63.6 | 26.9 | 9.5 |
| Changes in mood | 515 | 69.9 | 58.1 | 32.6 | 9.4 |
| Changes in memory | 517 | 68.86 | 58 | 36 | 6.1 |
| Changes in muscle tone | 513 | 62.38 | 53.7 | 35.4 | 10.9 |
| Drowsiness | 512 | 58.98 | 54.1 | 36.9 | 9 |
| Nausea | 513 | 58.87 | 40.2 | 38.5 | 21.2 |
| Change in taste | 514 | 57.98 | 40.2 | 35.1 | 24.7 |
| Pain | 515 | 57.48 | 53.9 | 38.5 | 7.7 |
| Dry Mouth | 511 | 52.64 | 33 | 35.9 | 31 |
| Loss of appetite | 515 | 52.23 | 55.9 | 34.7 | 27.8 |
| Pins and needles | 515 | 47.38 | 39.7 | 43.34 | 16.9 |
| Changes in behaviour | 511 | 44.03 | 50.2 | 37.8 | 12 |
| Fever | 514 | 43.97 | 45.4 | 33.8 | 43 |
| Neuropathic pain | 514 | 41.83 | 57.2 | 35.2 | 7.3 |
| Constipation | 512 | 41.60 | 37.6 | 50.3 | 12.2 |
| Headaches | 511 | 38.36 | 53.6 | 33.3 | 13.1 |
| Rash or itchy skin | 516 | 37.98 | 35.6 | 42.9 | 21.5 |
| Mouth ulcers | 513 | 37.04 | 48.3 | 28.2 | 23.6 |
| Diarrhoea | 514 | 34.82 | 47.3 | 38 | 14.7 |
| Breathlessness | 510 | 26.67 | 55.2 | 34.4 | 10.4 |
| Cough | 510 | 23.73 | 37.1 | 39.8 | 23 |
| Bleeding/ bruising | 512 | 23.63 | 17.2 | 52.7 | 30 |
| Anxiety | 513 | 23.39 | 60.8 | 31.3 | 7.8 |
| Insomnia | 515 | 22.91 | 65.2 | 29.5 | 5.2 |
| Vomiting | 511 | 22.90 | 44 | 37.4 | 18.7 |
| Loss of bladder control | 512 | 21.09 | 46.5 | 38.6 | 14.9 |
| Swallowing difficulties | 510 | 20.39 | 52.2 | 37 | 10.9 |
| Difficulty with urination | 510 | 17.06 | 46.2 | 43.6 | 10.3 |
| Seizures | 510 | 7.84 | 65.7 | 17.1 | 17.1 |
| Hiccups | 510 | 6.27 | 21.4 | 39.3 | 39.3 |

Supplemental Table 2: Ranking of acute and chronic symptoms by consumers. Numbers represent the number of consumers that ranked each symptom in that rank.

| **Symptoms** | **Acute** | | | | |  | **Chronic** | | | | |
| --- | --- | --- | --- | --- | --- | --- | --- | --- | --- | --- | --- |
|  | **Rank 1 (n=403)** | **Rank 2 (n=398)** | **Rank 3 (n=394)** | **Rank 4 (n=297)** | **Rank 5 (n=272)** |  | **Rank 1 (n=182)** | **Rank 2 (n=181)** | **Rank 3 (n=180)** | **Rank 4 (n=122)** | **Rank 5 (n=100)** |
| Fatigue | 47 | 40 | 37 | 28 | 29 |  | 8 | 12 | 12 | 16 | 9 |
| Insomnia | 42 | 42 | 32 | 20 | 11 |  | 18 | 19 | 12 | 11 | 8 |
| Changes in memory | 32 | 40 | 29 | 14 | 12 |  | 25 | 19 | 19 | 7 | 3 |
| Change in sex life | 28 | 13 | 18 | 13 | 26 |  | 16 | 9 | 17 | 10 | 17 |
| Changes in mood | 28 | 24 | 28 | 16 | 12 |  | 10 | 23 | 11 | 7 | 7 |
| Loss of appetite | 21 | 8 | 7 | 6 | 5 |  | 5 | 0 | 2 | 3 | 0 |
| Change in taste | 19 | 32 | 15 | 9 | 9 |  | 3 | 3 | 1 | 1 | 1 |
| Neuropathic pain | 16 | 12 | 8 | 9 | 5 |  | 7 | 7 | 3 | 3 | 1 |
| Pain | 16 | 13 | 15 | 17 | 13 |  | 4 | 8 | 5 | 8 | 4 |
| Breathlessness | 15 | 5 | 9 | 4 | 5 |  | 2 | 0 | 4 | 1 | 1 |
| Anxiety | 14 | 23 | 21 | 22 | 18 |  | 8 | 13 | 17 | 7 | 4 |
| Headaches | 13 | 7 | 14 | 8 | 2 |  | 9 | 0 | 2 | 1 | 0 |
| Nausea | 12 | 13 | 14 | 12 | 8 |  | 0 | 0 | 2 | 3 | 3 |
| Dry Mouth | 10 | 6 | 7 | 1 | 5 |  | 12 | 0 | 4 | 0 | 0 |
| Cough | 9 | 11 | 5 | 4 | 4 |  | 6 | 2 | 1 | 0 | 0 |
| Changes in muscle tone | 8 | 11 | 19 | 19 | 11 |  | 6 | 13 | 6 | 6 | 3 |
| Diarrhoea | 8 | 2 | 6 | 8 | 3 |  | 0 | 2 | 1 | 1 | 1 |
| Fever | 8 | 15 | 12 | 8 | 7 |  | 6 | 4 | 7 | 3 | 2 |
| Loss of bladder control | 8 | 9 | 8 | 10 | 9 |  | 2 | 2 | 4 | 3 | 3 |
| N/A | 7 | 15 | 21 | 16 | 20 |  | 11 | 16 | 20 | 14 | 15 |
| Other | 7 | 6 | 11 | 5 | 12 |  | 6 | 3 | 6 | 3 | 3 |
| Mouth ulcers | 6 | 15 | 13 | 11 | 7 |  | 3 | 2 | 3 | 0 | 0 |
| Seizures | 6 | 2 | 3 | 0 | 1 |  | 0 | 3 | 0 | 0 | 0 |
| Changes in behaviour | 4 | 3 | 9 | 2 | 5 |  | 3 | 3 | 7 | 3 | 5 |
| Constipation | 4 | 6 | 3 | 8 | 4 |  | 1 | 1 | 0 | 6 | 1 |
| Pins and needles | 3 | 7 | 7 | 8 | 13 |  | 6 | 4 | 4 | 2 | 4 |
| Vomiting | 3 | 0 | 1 | 3 | 0 |  | 0 | 0 | 0 | 0 | 0 |
| Bleeding/ bruising | 2 | 5 | 0 | 0 | 0 |  | 0 | 1 | 1 | 1 | 0 |
| Difficulty with urination | 2 | 5 | 2 | 2 | 1 |  | 1 | 4 | 2 | 0 | 1 |
| Drowsiness | 2 | 8 | 8 | 9 | 5 |  | 1 | 3 | 4 | 1 | 2 |
| Rash or itchy skin | 2 | 2 | 8 | 4 | 5 |  | 1 | 1 | 1 | 1 | 2 |
| Swallowing difficulties | 1 | 10 | 4 | 1 | 5 |  | 2 | 4 | 2 | 0 | 0 |
| Hiccups | 0 | 0 | 0 | 0 | 0 |  | 0 | 0 | 0 | 0 | 0 |

Supplemental Table 3: Prevalence and impact of symptoms reported by healthcare professionals in Round 1

| Symptom | N | Prevalence (%) | Major or Moderate impact (%) | Proportion of patients undermanaged (%) |
| --- | --- | --- | --- | --- |
| Fatigue | 109 | 98.2 | 98.9 | 91.2 |
| Anxiety | 109 | 98.2 | 96.8 | 84.6 |
| Anorexia | 109 | 91.7 | 91.6 | 89.0 |
| Insomnia | 109 | 89.9 | 88.4 | 80.2 |
| Neuropathic pain | 109 | 86.1 | 89.5 | 98.9 |
| Memory issues | 109 | 85.3 | 75.8 | 89.0 |
| Cachexia | 109 | 84.4 | 87.4 | 83.5 |
| Depression | 109 | 82.6 | 87.4 | 85.7 |
| Breathlessness | 109 | 80.7 | 82.1 | 82.4 |
| Sensory neuropathy | 109 | 77.1 | 76.8 | 85.7 |
| Drowsiness | 109 | 74.3 | 73.7 | 80.2 |
| Pain | 109 | 95.4 | 94.7 | 61.5 |
| Nausea | 109 | 95.4 | 93.7 | 62.6 |
| Constipation | 109 | 89.9 | 90.5 | 53.8 |
| Xerostomia | 109 | 75.2 | 65.3 | 73.6 |
| Vomiting | 109 | 74.3 | 82.1 | 56.0 |
| Dysgeusia | 109 | 71.6 | 68.4 | 81.3 |
| Diarrhoea | 109 | 69.7 | 81.1 | 60.4 |
| Sexual dysfunction | 109 | 67.9 | 65.3 | 87.9 |
| Cough | 109 | 65.1 | 69.5 | 72.5 |
| Mucositis | 109 | 63.3 | 78.9 | 73.6 |
| Dysphagia | 109 | 61.5 | 75.8 | 80.2 |
| Behaviour changes | 109 | 60.6 | 75.8 | 86.8 |
| Skin rash/itch | 109 | 59.3 | 68.4 | 67.0 |
| Fever/sweats | 109 | 56.9 | 61.1 | 65.9 |
| Headaches | 109 | 49.5 | 64.2 | 51.6 |
| Incontinence | 109 | 44.0 | 60.0 | 76.9 |
| Bleeding/ bruising | 109 | 44.0 | 51.6 | 68.1 |
| Difficulty with urination | 109 | 36.7 | 61.1 | 69.2 |
| Seizures | 109 | 30.3 | 61.1 | 40.7 |
| Hiccups | 109 | 21.1 | 40.0 | 62.6 |

**Note:** *Severity assessments were only completed by 95 healthcare professionals; and the proportion of patients undermanaged is based on n=91*
